# Evaluating Symptom Burden Among Omani Women Newly Diagnosed with Breast Cancer: A Cross-Sectional Study

**DOI:** 10.3390/curroncol32020059

**Published:** 2025-01-22

**Authors:** Kouthar Al Alawi, Amal Al Fahdi, Moon Fai Chan, Hana Al Sumri, Mohammed Al-Azri

**Affiliations:** 1 Department of Family Medicine and Public Health, College of Medicine and Health Sciences, Sultan Qaboos University, Muscat 123, Oman; s121343@student.squ.edu.om (K.A.A.); moonf@squ.edu.om (M.F.C.); alsumry@squ.edu.om (H.A.S.); 2 Department of Holistic Care, Psychosocial Unit, Sultan Qaboos Comprehensive Cancer Care and Research Center, University Medical City, Muscat 123, Oman; a.alfahdi@cccrc.gov.om

**Keywords:** breast cancer, chemotherapy, quality of life, Oman

## Abstract

Background: Breast cancer (BC) is the most common malignancy affecting women globally, significantly impacting their quality of life (QoL). This study aimed to assess the prevalence and severity of symptoms in newly diagnosed BC patients undergoing chemotherapy in Oman using the Edmonton Symptom Assessment System (ESAS-A); Materials and Methods: A cross-sectional study was conducted between December 2022 and February 2024 at the Sultan Qaboos Comprehensive Cancer Care and Research Centre (SQCCCRC), University Medical City, Oman. The study included 105 Omani women, aged 18 to 60, diagnosed with stage I to III BC and undergoing chemotherapy. Symptom evaluation was performed using ESAS-A. Descriptive statistics were employed to summarize socio-demographic characteristics and clinical outcomes, while the Mann–Whitney U test and multiple linear regression analysis were used to examine associations between independent variables and symptom scores; Results: Out of 127 invited participants, 105 (82.6%) agreed to participate. The average age was 43.6 years (SD = 7.2). Fatigue (37.1%), poor well-being (30.5%), and drowsiness (27.6%) were the most commonly reported symptoms. Anxiety and depression affected 21.9% and 17.1% of participants, respectively. Linear regression analysis showed that having children was linked to higher fatigue and shortness of breath, while inversely associated with pain. A family history of chronic disease was significantly correlated with higher depression scores; Conclusions: This study is the first in Oman to utilize ESAS-A for assessing symptom burden in newly diagnosed BC patients undergoing chemotherapy. The findings highlight the importance of personalized symptom management and enhanced supportive care to improve patient well-being.

## 1. Introduction

Breast cancer (BC) remains the most common cancer affecting women globally, making up 19.4% of all cancer cases and 34.7% of cancers in women [1]. While BC rates differ between populations, the number of cases has steadily grown over the last forty years, with a yearly increase of 0.5% between 2010 and 2019 [2]. Women facing a BC diagnosis often experience a wide range of symptoms, not only from the disease itself but also from the treatments. These symptoms can profoundly impact their quality of life (QoL), sometimes resulting in treatment delays or even discontinuation [3,4].

Physical symptoms such as pain, fatigue, nausea, and swelling of the lymph nodes can lead to significant discomfort and impair daily functioning [5]. Psychological challenges, including anxiety, depression, and emotional distress, further increase the burden, negatively affecting mental health and overall well-being [6]. Social and functional difficulties may also emerge, such as changes in body image, sexual dysfunction, and social isolation, all of which strain personal relationships and diminish social support networks [7]. Additionally, cognitive impairments, including memory problems and difficulties with concentration, further degrade QoL [8]. These combined effects underscore the critical need for comprehensive symptom management and supportive care to improve the overall QoL for women with BC.

Women diagnosed with BC in Oman experience significant psychosocial distress, including anxiety, depression, and a diminished sense of identity, all of which deeply impact their QoL [7]. Many turn to various coping strategies to accept their diagnosis and find new meaning, often viewing their illness as part of God’s will [9,10]. Additionally, they face physical side effects from BC treatment, such as fatigue, pain, and changes in appearance, which adversely affect their physical functioning, daily activities, and overall QoL [9]. These challenges frequently require substantial adjustments in both their personal and professional lives.

The ESAS is a widely used clinical tool designed to assess the symptoms experienced by patients with chronic diseases and cancers, including BC [11]. It evaluates QoL by measuring key symptoms such as pain, fatigue, drowsiness, appetite loss, shortness of breath, nausea, anxiety, depression, and overall well-being [12]. Originally developed and validated for palliative care settings, the ESAS is essential in managing symptoms for patients with advanced cancer, particularly in monitoring and addressing chemotherapy-related symptoms during treatment [13,14].

Several studies have explored the use of ESAS in BC care, consistently demonstrating its effectiveness in predicting patient outcomes and enhancing both symptom management and overall care quality [15,16]. For instance, research has demonstrated that ESAS can reliably predict patient outcomes, such as time to death, by evaluating symptoms like loss of appetite, which has been linked to reduced survival in BC patients [15]. In 2006, ESAS was integrated into Cancer Care Ontario’s Provincial Palliative Care Integration Project to enhance cancer care for palliative patients through the regular use of symptom assessments [16]. This assessment tool has been extensively discussed in the literature and translated into more than 20 languages globally, including Arabic [14,17,18]. The English version of ESAS demonstrated a Cronbach’s alpha coefficient of 0.79, indicating strong internal consistency [19]. This study aims to evaluate the prevalence and severity of symptoms—such as pain, fatigue, drowsiness, nausea, appetite loss, shortness of breath, depression, anxiety, poor well-being, and other health issues—among newly diagnosed BC patients undergoing chemotherapy, utilizing the Arabic version of the ESAS-A.

## 2. Materials and Methods

### 2.1. Tool Used for Data Collection

The Arabic version of the ESAS-A is a culturally tailored instrument specifically developed for Arabic-speaking patients [14]. The ESAS-A demonstrates high reliability and validity in assessing symptom severity among cancer patients, with an average Cronbach’s alpha of 0.84, reflecting strong internal consistency [14]. Research conducted in Arabic-speaking countries, including Jordan, has confirmed the effectiveness and accuracy of the ESAS-A in evaluating symptoms in patients with advanced cancer [20]. The ESAS-A evaluates ten symptoms: pain, fatigue, drowsiness, nausea, appetite loss, depression, anxiety, feelings of poor well-being, and other health problems. Each symptom is rated on a scale from 0 to 10, where 0 indicates the absence of the symptom and 10 represents the highest severity [14].

### 2.2. Study Design, Setting, and Patient Recruitment

The Sultan Qaboos Comprehensive Cancer Care and Research Centre (SQCCCRC), University Medical City, is a newly established, government-operated institution in Muscat, the capital of Oman. This facility offers a comprehensive range of treatment options for cancer patients across the country, including surgical procedures, radiotherapy, chemotherapy, hormonal therapies, and palliative and psychological care. SQCCCRC, University Medical City, provides extensive support for individuals diagnosed with various types of cancer, including BC.

A cross-sectional study was conducted at the Sultan Qaboos Comprehensive Cancer Care and Research Centre (SQCCCRC), University Medical City, from December 2022 to February 2024. The study cohort comprised Omani women aged 18 to 60 diagnosed with BC who were receiving chemotherapy in the daycare units. All patients were assessed uniformly during their first chemotherapy session, ensuring consistency in timing and reducing variation in the data collection process. Patients included in the study were diagnosed with cancer stages ranging from stage one to stage three. Women with stage four BC were excluded to avoid potential biases arising from differences in symptoms and treatment experiences compared to earlier stages. Hormonal receptor (ER/PR) and HER2 status information were not available for this study, and this limitation has been acknowledged.

Participants were given a comprehensive information sheet detailing the study’s objectives and a consent form for those who chose to participate. They were assured that their involvement was entirely voluntary and would not affect their medical care at the SQCCCRC, University Medical City. Participants were assured that all collected data would remain confidential and be utilized solely for research purposes. Non-Omani women and those with substantial psychiatric disorders or cognitive impairments were identified through medical records and excluded from the study. While data on specific logistical challenges for rural patients were not collected, the inclusion of participants from rural areas highlights the broader context of healthcare access within Oman. The first author administered the questionnaires, clarifying the study’s objectives and the scaling process. For participants unable to read or write, the first author verbally administered the questions and documented their responses accordingly.

### 2.3. Sample Size Calculation

The sample size required for this study was calculated based on the data regarding depressive symptoms obtained from the ESAS, utilizing a cross-sectional study design. A previous study conducted in Jordan reported a depression prevalence rate of 30.2% among patients diagnosed with BC [21]. Assuming a variation of ±9% from this expected prevalence, it was determined that a sample size of 97 participants would be necessary to achieve a significance level of 5%. These calculations were carried out using the online EPITOOLS 2025 software [22].

### 2.4. Statistical Analysis

Data analysis was conducted with the Statistical Package for the Social Sciences (SPSS), version 23.0 (IBM Corp., Armonk, New York, NY, USA). A significance level of 5% was established for all analyses. Descriptive statistics, including percentages and means, were employed to summarize the participants’ socio-demographic characteristics and clinical outcomes. The ESAS scale was utilized to assess various symptoms, where a score of 0 indicated the absence of the symptom and a score of 10 represented the most severe manifestation of the symptom. The Mann–Whitney test was employed to explore the association between independent variables and ESAS scores. All statistical tests were two-sided, with *p* values less than 0.05 considered statistically significant. A multiple linear regression model was implemented to identify the most significant variables associated with each dimension of the ESAS. The study received approval from the local medical research ethics committee of the SQCCCRC, University Medical City (IRB & EC Project ID: CCCRC-20-2022) approved on 15 August 2022.

## 3. Results

### 3.1. Demographic and Clinical Characteristics

A total of 127 women with BC were invited to participate in the study, and 105 agreed, with a response rate of 82.6%. The women’s mean age was 43.6 years (SD = 7.2), where the majority (73.3%) were within the age range from 40 to 60 years. Most women were married (84.8%), educated and could read and write (76.2%), had children (89.5%), and were unemployed or retired (63.8%), and a large proportion had a family history of cancer (44.8%) or chronic diseases (41.9%). Additionally, 70.5% were diagnosed at an early stage (I and II) and lived in rural/village areas (61.9%), with 86.7% having sufficient financial support and 93.3% expressing satisfaction with family relationships (Table 1).

### 3.2. Symptom Prevalence and Severity

The most prevalent reported symptom was tiredness (37.1%), with a mean score of 2.50 (SD = 2.6), followed by poor well-being (30.5%) with a mean score of 2.2 (SD = 2.8), and other health problems (27.6%) with a mean score of 2.0 (SD = 2.4). Other symptoms included drowsiness (27.6%, mean score 1.9, SD = 2.2), anxiety (21.9%, mean score 1.7, SD = 2.5), pain (19.0%, mean score 1.7, SD = 2.1), depression (17.1%, mean score 1.1, SD = 1.9), loss of appetite (13.3%, mean score 1.3, SD = 2.3), nausea (7.6%, mean score 0.9, SD = 1.8), and shortness of breath (7.6%, mean score 0.82, SD = 1.6) (Table 2).

### 3.3. Analysis of ESAS-A Scores

The analysis of the total score dimension of ESAS-A concerning various demographic and clinical variables showed that having children was associated with significantly lower total ESAS-A scores (*p* = 0.012), with those without children reporting a higher median score (28.50) compared to those with children (11.00) (Table 3).

### 3.4. Linear Regression Analysis

Linear regression analysis was conducted to examine the relationship between demographic and clinical variables and symptoms, as evaluated by the ESAS-A. The analysis revealed that having children showed a significant negative association with the symptoms of pain (B = −1.686, *p* = 0.035). Conversely, having children was significantly associated with higher levels of tiredness (B = −2.662, *p* = 0.005), increased shortness of breath (B = −1.255, *p* = 0.037), and an increase in other health problems (B = −1.793, *p* = 0.043). Additionally, the analysis showed that having a family history of chronic disease was significantly and positively associated with depression (B = 0.817, *p* = 0.035). For other health problems, employment status showed a marginally significant negative association (B = −0.853, *p* = 0.089), indicating that unemployed or retired patients tended to report higher levels of these issues (Table 4).

## 4. Discussion

To the best of our knowledge, this is the first study conducted in Oman that used the ESAS-A specifically with newly diagnosed BC women undergoing chemotherapy treatments. The average age of the participants, ranging between 40 and 60 years, is consistent with previous studies suggesting that this age group commonly bears family responsibilities [23]. However, no significant associations were found between marital status or education level and the overall symptom burden assessed by the ESAS-A, which aligns with findings from similar studies [24].

In our study, 35.2% of patients were employed, while 63.8% were unemployed or retired. Employment status and the presence of children were both identified as significant predictors of other health problem scores. Specifically, unemployment or retirement) and having children were associated with higher scores in this dimension [25]. The psychological impact of unemployment or financial insecurity can be substantial. Financial difficulties can lead to higher levels of psychological distress and impact adherence to treatment regimens [26]. Research indicates that individuals facing financial difficulties often experience elevated levels of psychological distress, which can hinder their ability to cope with the disease and adhere to treatment regimens [27]. Nonetheless, a significant number of patients in the study (93.3%) reported satisfaction with their family relationships, underscoring the crucial role of familial support in managing chronic diseases such as cancer. Previous studies have consistently shown that strong family support is associated with better psychological outcomes and improved treatment adherence in cancer patients [28,29].

Nearly half of our patients (44.8%) had a family history of cancer, while a considerable portion (41.9%) reported a family history of chronic disease. This finding underscores well-established evidence highlighting the increased risk associated with a family history of cancer [30]. The presence of a family history of BC can influence mental well-being, affecting coping mechanisms, emotional distress, and overall psychological adaptation within the family unit [31]. Individuals with a family history of BC, compared to those without, often perceive themselves to be at a higher risk of developing the disease, which can lead to anxiety, stress, and a constant fear of diagnosis, negatively impacting their mental health [32].

Indeed, a family history of other chronic diseases in our study emerged as a significant predictor of depression scores, as participants may feel more vulnerable to health issues in general and experience uncertainty about their future health, which increases psychological distress [33]. In line with the findings of prior studies, individuals with chronic diseases face an elevated risk of poor mental health outcomes. For instance, a recent study analyzing the Behavioral Risk Factor Surveillance System (BRFSS) found that individuals with chronic diseases such as kidney disease, coronary heart disease, asthma, and cholesterol disorders experienced significantly longer durations of poor mental health. These results underscore the importance of addressing the psychological impact of chronic illnesses to enhance overall well-being and treatment outcomes [34]. Consistent with prior studies, individuals with chronic diseases are at an increased risk of experiencing poor mental health outcomes. For example, a recent analysis of the Behavioral Risk Factor Surveillance System (BRFSS) revealed that people with chronic conditions, including kidney disease, coronary heart disease, asthma, and cholesterol disorders, reported significantly longer periods of poor mental health. These findings highlight the necessity of addressing the psychological effects of chronic illnesses to improve overall well-being and treatment results. In contrast, factors such as family history of cancer and time since diagnosis were not significant predictors of depressive symptoms in our study, even though they were identified as relevant in the previous literature [35,36].

The lack of significance associated with diagnosis time may suggest that psychological distress is more closely tied to the overall experience of breast cancer and the treatment burden rather than the timing of the diagnosis itself. While earlier research indicates that delayed diagnosis can worsen anxiety and depression, our study included only patients at stages I to III. This may explain the lack of significance for diagnosis time, as women in these earlier stages might perceive their disease as more manageable, leading to lower psychological impacts compared to those with more advanced cancer stages.

Similarly, although a family history of cancer has been correlated with increased anxiety and depression in some studies, it did not prove significant in our analysis. This might be attributable to other unmeasured factors, such as familial coping strategies, cultural attitudes towards illness, or the availability of social support. Moreover, a strong family support system, reported by 93.3% of participants in this study, may have mitigated the psychological effects associated with a family history of cancer.

It is important to note that cancer stage, which has been linked to heightened psychological distress in earlier research, was not examined in our study beyond stage III. We intentionally excluded stage IV patients to minimize symptom variability and maintain homogeneity among our cohort. However, this may limit our findings’ comparability to studies that include patients with advanced cancer stages. Future research should encompass a wider range of cancer stages and investigate additional clinical and psychosocial factors that may influence depression and other symptoms. Despite these limitations, our findings emphasize the importance of addressing the psychological impact of chronic diseases and other factors affecting mental health outcomes in breast cancer patients. Implementing targeted mental health interventions, such as counseling and psychosocial support, into standard cancer care is crucial for enhancing patients’ overall quality of life.

Most of our patients (61.9%) resided in villages, while 38.1% lived in the capital city. This distribution suggests potential geographic disparities in access to cancer healthcare services, with a concentration of services in the capital city. This underscores the need for outreach and support programs in rural areas to ensure equitable cancer care. Although our study did not find significant differences between the geographic location of patients and their ESAS-A scores, other studies have highlighted that patients living in rural areas often encounter several barriers, including limited access to specialized cancer care, longer travel times, and fewer healthcare resources [26,37]. Moreover, the availability of psychosocial support services, such as counseling and support groups, is often limited to rural areas, and continuity of care may be compromised due to the scarcity of healthcare providers [38]. These barriers can lead to delays in cancer diagnosis and treatment, ultimately affecting survival rates and quality of life [39].

The most commonly reported symptom by participants in the ESAS-A was tiredness (37.1%), while the least reported was nausea (7.6%). Tiredness in cancer patients can be attributed to various factors, including pain, anemia, tumor cachexia, and the side effects of cancer-related treatments [40,41]. Additionally, the psychological burden of cancer, such as fear of recurrence, treatment stress, uncertainty about outcomes, body image issues, sexual problems, and social difficulties, can lead to exhaustion and persistent tiredness [40]. On the other hand, the finding of a low level of nausea in our study contrasts with the widely recognized prevalence of chemotherapy-induced nausea, a significant and distressing symptom in cancer patients, with reported prevalence rates ranging from 20% to 50%, and needs further investigation [42].

Participants with children experienced significantly lower scores on the ESAS-A compared to those without children, suggesting a lower overall symptom burden. This finding may reflect the emotional and psychological support provided by familial bonds, which could serve as a protective factor against worsening symptoms. Previous research has suggested that strong familial connections and caregiving roles may enhance resilience and provide a sense of purpose, potentially mitigating psychological distress and improving symptom management [43]. However, it is important to note that parenting responsibilities may still pose challenges in balancing caregiving with personal health needs, which could warrant further exploration in future studies.

The balance between treatment, recovery, and parenting can be overwhelming, affecting not only their physical health but also their emotional and psychological well-being [44]. Additionally, they may feel guilty or anxious about not being able to fulfill their parental duties to the desired extent. [43]. On the other hand, the presence of children in our study showed a negative association with pain scores. However, a previous study reported that women with children diagnosed with cancer experienced greater pain compared to women without children [33]. It has been found that having children might provide emotional support, which can alleviate some psychological burdens and potentially reduce feelings of pain and tiredness [45].

While specific studies directly linking parenthood to reduced shortness of breath in cancer patients are limited, it is well-established that social support and psychological well-being can significantly influence physical symptoms. Emotional support and a sense of purpose—often derived from parenting—have been shown to improve overall quality of life and enhance symptom management in cancer patients [46]. The observed associations between parenthood and lower fatigue and reduced shortness of breath scores in our study suggest several possible explanations. Parenthood may contribute to improved psychosocial support and coping mechanisms, thereby alleviating symptoms such as fatigue and respiratory distress [47]. Additionally, lifestyle factors associated with caregiving responsibilities, including structured routines and increased physical activity, could positively impact symptom management [47].

Our study has several limitations. First, Although the study was conducted at a single oncology center (SQCCCRC), University Medical City, we believe this should not affect the generalizability of the findings to broader populations across Oman, as patients treated at the center come from various regions of the country. Second, the cross-sectional design of our study restricts our ability to establish causal relationships between variables. Future research should prioritize the evaluation of tailored symptom management interventions, such as psychological support programs. Finally, comparative studies examining different cancer types or stages within Oman could further elucidate specific symptom profiles and inform targeted interventions based on disease characteristics.

## 5. Conclusions

This study marks the first use of the ESAS-A in Oman to evaluate symptom prevalence and severity among newly diagnosed BC patients undergoing chemotherapy. The findings indicate that tiredness was the most frequently reported symptom, followed by poor well-being, other health problems, drowsiness, anxiety, pain, depression, loss of appetite, nausea, and shortness of breath. Analyzing symptoms in relation to demographic and clinical factors revealed that participants with children reported fewer overall symptoms but experienced higher levels of tiredness, shortness of breath, and other health problems. Conversely, patients without children reported a greater overall symptom burden. Additionally, a family history of chronic disease was associated with increased depression, while unemployment or retirement correlated with a higher incidence of other health problems. These results highlight the need for comprehensive symptom management and the development of tailored supportive care strategies to improve the overall well-being of BC patients in Oman.

## Figures and Tables

**Table 1:** Demographic and clinical characteristics of patients with BC (*n* = 105).

Characteristic/Outcome	*n* (%)
**Age** ^	
18–39	27 (25.7)
40–60	77 (73.3)
**Marital status**	
Single	16 (15.2)
Married	89 (84.8)
**Education level** ^	
Illiterate	24 (22.9)
Educated	80 (76.2)
**Employment** ^	
Employed	37 (35.2)
Unemployed/retired	67 (63.8)
**Have children** †	
Yes	94 (89.5)
No	8 (7.8)
**Family history of cancer**	
Yes	47 (44.8)
No	58 (55.2)
**Family history of chronic disease**	
Yes	44 (41.9)
No	61 (58.1)
**Relationships with family members**	
Satisfied	98 (93.3)
Unsatisfied	7 (6.7)
**Diagnosis time**	
Early	74 (70.5)
Late	31 (29.5)
**Financial Support** †	
Yes	91 (86.7)
No	11 (10.5)
**Location**	
Capital city	40 (38.1)
Village	65 (61.9)

^ 1 missing; † 3 missing.

**Table 2:** Description of ESAS symptoms (*n* = 105).

Symptoms on ESAS	Mean (SD)	Median [Q1–Q3]	*n* (%) of Moderate to Severe Symptoms
Pain	1.70 (2.153)	1.0 [0.0–3.0]	20 (19.0)
Tiredness	2.50 (2.606)	2.0 [0.0–5.0]	39 (37.1)
Drowsiness *	1.95 (2.209)	1.0 [0.0–4.0]	29 (27.6)
Nausea	0.94 (1.860)	0.0 [0.0–1.5]	8 (7.6)
loss of appetite	1.37 (2.394)	0.0 [0.0–2.0]	14 (13.3)
Shortness of Breath	0.82 (1.616)	0.0 [0.0–1.0]	8 (7.6)
Depression	1.16 (1.967)	0.0 [0.0–1.0]	18 (17.1)
Anxiety *	1.76 (2.506)	1.0 [0.0–3.0]	23 (21.9)
Poor Well-being *	2.29 (2.888)	1.0 [0.0–5.0]	32 (30.5)
Other Health Problems	2.03 (2.455)	1.0 [0.0–4.0]	29 (27.6)

* 1 missing.

**Table 3:** Total score dimension (ESAS) (*n* = 105).

Variable	*n* (%)	Total Score Median [Q1–Q3]	*p*-Value
**Age** ^			0.577
18–39	27 (25.7)	13.00 [6.00–27.00]	
40–60	77 (73.3)	12.00 [2.50–29.00]	
**Marital status**			0.369
Single	16 (15.2)	20.50 [2.50–30.50]	
Married	89 (84.8)	12.00 [3.00–28.00]	
**Education level** ^			0.728
Illiterate	24 (22.9)	9.00 [2.25–31.00]	
Educated	80 (76.2)	13.00 [4.00–28.00]	
**Employment** ^			0.582
Employed	37 (35.2)	14.00 [3.50–28.00]	
Unemployed/retired	67 (63.8)	11.50 [2.25–28.25]	
**Have children** †			**0.012** *
Yes	94 (89.5)	28.50 [24.50–34.75]	
No	8 (7.8)	11.00 [3.00–27.25]	
**Family history of cancer**			
Yes	47 (44.8)	13.50 [4.50–30.25]	0.696
No	58 (55.2)	11.00 [3.00–28.00]	
**Family history of chronic disease**			
Yes	44 (41.9)	17.00 [5.00–31.00]	0.188
No	61 (58.1)	10.00 [3.00–27.00]	
**Relationship with family**			
Satisfied	98 (93.3)	12.00 [3.00–28.00]	0.363
Unsatisfied	7 (6.7)	24.00 [11.00–28.00]	
**Diagnosis time**			
Early	74 (70.5)	11.00 [5.00–29.00]	0.617
Late	31 (29.5)	12.50 [1.75–24.50]	
**Financial support** †			
Yes	91 (86.7)	11.00 [3.00–28.00]	0.170
No	11 (10.5)	25.00 [6.00–33.00]	
**Location**			
Capital	40 (38.1)	14.00 [3.00–28.00]	0.825
Village	65 (61.9)	11.00 [3.00–27.00]	

* Significant at *p* < 0.05; ^ 1 missing; † 3 missing; Mann–Whitney test was used to assess the association.

**Table 4:** Linear regression analysis for variables associated with ESAS symptoms (*n* = 105).

Model	Unstandardized Coefficient (B)	Standard Error	Standardized Coefficient (Beta)	t-Value	*p*-Value	95.0% Confidence Interval for B		Collinearity Statistics VIF
**Pain**								
(Constant)	3.250	0.755		4.302	0.000	1.751	4.749	
Having Children	−1.686	0.787	−0.210	−2.143	**0.035** *	−3.248	−0.125	1.000
**Tiredness**								
(Constant)	4.875	0.891		5.473	0.000	3.108	6.642	
Having Children	−2.662	0.928	−0.276	−2.869	**0.005** *	−4.503	−0.822	1.000
**Shortness of Breath**								
(Constant)	2.000	0.570		3.509	0.001	0.869	3.131	
Having Children	−1.255	0.594	−0.207	−2.114	**0.037** *	−2.433	−0.077	1.000
**Depression**								
(Constant)	0.003	0.575		0.005	0.996	−1.137	1.143	
Family history of chronic disease	0.817	0.382	0.206	2.135	**0.035** *	0.058	1.575	1.000
**Other health problems**								
(Constant)	4.779	1.121		4.262	0.000	2.554	7.005	
Employment	−0.853	0.497	−0.169	−1.715	0.089	−1.840	0.134	1.009
Have Children	−1.793	0.876	−0.201	−2.046	**0.043** *	−3.532	−0.054	1.009

* Significant at *p* < 0.05; VIF, Variance Inflation Factor.

## Data Availability

The data supporting the findings of this study are available from the corresponding author upon reasonable request. However, due to privacy/ethical restrictions, the data are not publicly available.
